# Epidemic Cerebro-Spinal Meningitis

**Published:** 1873-06

**Authors:** Edward Montgomery


*Epidemic Cerebro-Spinal Meningitis.* By EDWARD MONTGOMERY, M.D.

This disease, under different names, has prevailed to a considerable extent, both in this country and in Europe, since the beginning of the present century. From the descriptions given by various writers of the fourteenth, fifteenth, sixteenth and seventeenth centuries, it is probable that it visited different parts of the old world, but of this we are somewhat uncertain, because of the absence of a very minute and comprehensive clinical history of the cases, and for the lack of post-mortem examinations. "The first well-authenticated epidemic of the disease in this country occurred in 1806, from which time until 1816 it was constantly epidemic, particularly in the New England States, upon which it seemed to fasten." (*Clymer.*) From 1806 until the present time, partial epidemics and sporadic cases occasionally occurred both in the old and in the new world. In 1845 and 1846 it broke out in Illinois, and was there called "black tongue." In 1847 it prevailed in Mississippi, Tennessee, Missouri and Arkansas, and during our late civil war it occurred occasionally in both armies.

(*Clymer.*) In 1846, a very severe epidemic of it broke out in the city of Dublin, Ireland, where it principally prevailed in the work-houses and hospitals, although there were many fatal cases in healthy parts of the city, and where many fell victims to its virulence whose surroundings were unexceptionable.

Dr. Simon, of London, a very high authority, says, that "soldiers in barracks, the inmates of jails and poor-houses, the inhabitants of crowded, filthy streets, and every place where filth and bad ventilation prevail, are its favorite resorts." But in this country we know that it has devastated little hamlets in the prairies, fastened its unrelenting grasp on the scattered denizens of our newly-settled districts, and prevailed for years together in the healthiest portions of Pennsylvania and Massachusetts. "Notwithstanding its wide geographical distribution, there is no corresponding diffusion amongst any one population during its prevalence, it, as a rule, being limited, during an outbreak, to certain localities, and to certain portions of the population of such localities. The distribution of the disorder seems to be by a series of isolated eruptions, rather than by general spreading." (*Clymer.*)

This is one of its strange peculiarities and anomalies; it will break out in certain neighborhoods, and in certain families in such neighborhoods, without our being able to discover any cause for such predilection. Sometimes it will prostrate a whole family at once, and again carry off one in a large family and the rest all escape; in this respect differing very much from cholera, influenza, or small-pox. Like cholera and influenza it seems to be caused by some toxic principle in the atmosphere, and by it carried to different portions of the world; but unlike those epidemics, it does not appear to spread by contagion or infection; neither will the effluvia, emanations, or excrements of the sick, propagate the disease, as I have abundant evidence of the fact; often have I witnessed the small, close chamber, where a patient was dying, crowded with the neighboring children, and yet none of them becoming infected with the disease. Another peculiarity of the disorder is its varying symptomatology. In 1847, when it prevailed in Missouri, Mississippi, Tennessee and Arkansas, the fauces, throat and respiratory organs were implicated to such an extent that it was called "black tongue," "typhoid pneumonia," etc. In 1846, in Dublin, the cases had nearly all an extensive purpuric eruption, so that Dr. Stokes and others termed it purpuric fever; whilst in other epidemics, in other parts of the world, the type of the disease seemed of a congestive character, and so suddenly fatal, that it was called the "plague," the "*Malum Egyptiacum*," etc. And again, as in the present epidemic in this city, the spine and brain symptoms so predominate that the term cerebro-spinal meningitis seems alone to be appropriate, and to be generally employed. The term "spotted fever" has also been given to it in different parts of this country, but all these names belong to the same disease, manifesting itself under different types and forms, nearly all exhibiting the same morbid anatomy—a congestion of blood in the expanded vessels of the cerebro-spinal system, with more or less effusion and exudation of serum, semi-organized lymph or pus, and sometimes softening of portions of the brain and cord. It is true that Dr. Woodward, Chief of the U. S. Army Medical Bureau, says that many of the cases reported by different army surgeons, during the war, to his office, presented no signs of inflammation or structural lesion of any kind in the brain, cord, or membranes, and that this exemption from morbid changes in those parts, is not to be accounted for on the supposition that death had occurred too soon for those pathological appearances to be developed, since this freedom from structural change was also observed in cases where death had not ensued suddenly. But, on the whole, it is sufficiently evident from the signs and symptoms during life, and from the post-mortem evidences, that the cerebro-spinal system is the part principally involved, or at least that it is there wherein the disease most forcibly manifests itself. According to Stille, Clymer, Condie, Hirsch, Wunderlich, Neimeyer, Mayne and others, the morbid anatomy of the disease consists in an expansion of the vessels of the cerebro-spinal system, a superabundant quantity of blood in those vessels; this congestion and vascularity is especially seen in the pia mater, and when the patient has died early in the disease; an increased amount of clear, turbid or flocculent serum in the sub-arachnoid space, and in the ventricles; even in cases of the briefest duration there is an exudation of thick, pasty, semi-organized lymph, of a greenish-yellow color, on the base of the brain and medulla oblongata; the membranes at this stage are dry, and sometimes adherent to the brain or to each other. The cerebro-spinal fluid is increased, and portions of both brain and cord are found occasionally softened. When the disease has lasted for some time, the cerebral exudation is soft, opaque and yellowish, two or three lines in thickness, and may be compared to soft butter spread over the brain; or it may be denser, and have a pseudo-membranous look. It is found chiefly along the course of the vessels, and small in quantity, limited to and ramifying with them, or it may be in little irregular patches of variable size scattered over the brain surface, or covering the pons or medulla oblongata or cerebellum or parts of the cerebrum, or it may completely envelop the cerebrum, cerebellum and intracranial cord. Commonly superficial, it may dip down with the pia mater among the convolutions, or be found in the ventricles, or hanging and thickening, with an opaque, greenish pus, the serous fluid of the whole cavity. Purulent infiltration of the choroid plexus, and superficial softening of the walls of the ventricles, have been also observed. (*Clymer.*)

Pus, or purulent lymph, has also been found in the spinal canal, as well as in the base of the brain, in cases which have been somewhat tedious in their course. In nearly all the cases the expanded cerebro-spinal vessels have been found full of this black colored blood, and in some cases, fibrinous clots have been found in the heart after death. (*Stille.*)

As before intimated, this disease presents different forms or types in different epidemics, in different countries and even in the severe epidemic, and among the same class of patients. In some cases it is ushered in without any appreciable premonitory symptoms whatever, the patient being suddenly stricken down as by some fell poison, becomes delirious, tosses about for a few hours, tries to vomit every few minutes, with sighing and slow respiration, the skin dusky, the eyes suffused and sunken, and death by coma in ten to twenty-four hours. Occasionally these fulminant, blasting, or siderant cases, as they are variously termed, are accompanied with profuse diarrhœa, vomiting, and convulsions.

The tetanic form of the disease is more tedious in its progress, and of course more amenable to treatment; it is generally ushered in with chilliness, nausea, headache, frequently a general hyperæsthesia, or pains in some or all of the joints, sometimes sore throat, with pains in the ears; although there is some thirst, and the mouth is dry, but sparing drinks will be indulged in, the patient soon becomes much worse, there will be great jactitation, the cephalalgia will become more intense, the eyes will become suffused and injected, the face flushed, the head drawn back, the pulse quick, the bodily temperature from 100° to 103° F.; a trembling of the extremities when the patient is moved, intolerance of light, pupils contracted at first, but when exudation has taken place the pupils may be dilated or irregular, and the pulse or temperature may be abnormally reduced. In some of the cases an erythematous, eczematous, or purpuric, a morbillous or herpetic eruption may appear; but an eruption of any kind is not present in one-third of the cases, hence the misnomer of spotted or purpuric fever. There is often great deafness, apathy, and in all cases early prostration. When the case is severe the patient is terribly racked with the cephalalgia and pain in the throat and upper part of the spine from the tetanic contractions; but a few minutes' rest at a time can be indulged in, the patient suddenly waking up with piercing cries or low moans, and talkative delirium, or convulsions, or complete coma, may close the terrible sufferings. In tedious cases partial paralysis sometimes occurs, and the deafness occasionally remains permanent. Sometimes, also, vision is permanently impaired, and strabismus may result from the attack.

Dr. Kempf, in writing about a severe epidemic of this disease which occurred in the spring of 1863, in Dubois, Spencer and Perry counties, Illinois, describes three forms, which he names respectively, cerebro-spinal asphyxia, cerebro-spinal inflammation, and cerebro-spinal irritation; the first variety, he says, carried off the patients in many instances in a few hours, and no treatment seemed to have any effect. These fulminant cases were ushered in with chills, vomiting, diarrhœa, violent headache, soon followed by lethargy and extreme prostration, complete apathy, stupor as if profoundly intoxicated, the eyes suffused and injected, the skin covered with dark crimson or purple spots, coma and death. The inflammatory, or tetanic form, was also ushered in with chilliness, vomiting, headache, suffused countenance, injected eyes, apathy and debility. In a short time the head was retracted, the throat and neck painful, the petechial or purpuric spots appeared, and if not cured, delirium, convulsions, or coma, closed the scene. For these cases the treatment was bleeding, purging, plenty of calomel, veratrum viride, quinine, camphor, and opium. The doctor says he thinks bleeding and counter-irritation do harm, but the other remedies he employed seemed to save some cases. For the third variety—the cerebro-spinal irritation—morphine and quinine were all that were necessary to insure convalescence. Dr. McVey, of Morgan county, Illinois, reports his treatment for the disease as it appeared in 1863 and 1864 in that section of the State: it consisted in a moderate use of aperients and alteratives, friction to the spine and extremities, large doses of opium, four or five grains of opium with six or eight drops of Fowler's Arsenical Solution every four hours, blisters to the back of the neck, and if the cases were prolonged he also gave strychnine. He says this treatment proved rather successful, but we apprehend very few physicians would venture on such a free use of opium in this disease. Dr. Brown, in the same region of country, also reports his meeting with much success from similar treatment. (*Transactions of Illinois Medical Society.*) Dr. Liddell reports some cases treated by him in the Washington City Hospital, in 1864, in which his favorite remedy was opium, and he attempts to account for the beneficent action of this drug on the ground that it has the effect of preventing effusion from serous membranes. In one of Dr. Liddell's reported cases the patient was seized with the disease whilst taking quinine for an attack of ague, from which he was convalescing. This does not speak well for the remedial virtues of quinine in cerebro-spinal meningitis.

In the April number of the *American Journal of the Medical Sciences* for the year 1864, there is an article by Dr. Burns, on this disease as it occurred in the vicinity of Philadelphia, in which he advocates leeches, venesection, frictions, blisters, purgatives and quinine. He reports the particulars of some very alarming cases which almost miraculously recovered under this antiphlogistic treatment. If we examine the pages of the *Medical Press and Circular* of Dublin for 1867, we will find many cases exact counterparts of those of Dr. Burns which died under a stimulating treatment. Dr. Black, of Newark, Ohio, in the same journal, takes the opposite ground, and protests against antiphlogistics in all cases, and affirms his belief in the efficacy of tonics only. Indeed, in looking over the medical journals for 1863, '64, '65 and '66, although epidemics prevailing in different sections of the country during those years are presented to us with great uniformity of description, the same vivid picture of the malady being almost exactly reproduced in every instance, there is much contradiction and discrepancy with regard to the treatment.

Professor Wunderlich describes the disease as it appeared in Germany in 1863; its symptomatology and morbid anatomy as given by him is a counterpart of that delineated by numerous observers in this country. His treatment consisted in local depletion in sthenic cases, ice to the head, but not to the spine as there he observed it did more harm than good. Morphine, he says, allayed the pain in one case and it finally recovered, but chloroform inhalations and as a liniment was also used in the same case. Iodide of potassium was given in three cases which terminated in cure; he therefore recommends it, especially in protracted cases. He says there was no evidence of the disease being contagious or infectious.

Dr. Condie, in his report of the diseases of Philadelphia county for the year 1864, in the transactions of the Pennsylvania State Medical Association, treating of this malady, says that for the algid form he had tried alcoholic and diffusible stimulants with quinine, and externally used liniments, sinapisms, frictions, blisters, hot baths, etc., but without being convinced of their having the least good effect on the disease. For the tetanic form he chiefly relied on opium and quinine internally, and on frictions, liniments, blisters and dry cups along the spine, especially to the upper portion. An infusion of half an ounce each of cimicifuga, wild cherry bark, serpentaria and sassafras bark in a quart of boiling water; a wineglass of this infusion every three hours seemed also to do much good.

Dr. Githens, in his report of the cases treated by him in 1866 and 1867, in the Philadelphia Hospital, says that his principal measures consisted in ice to head, blisters to nuchæ, veratrum viride, carbonate or muriate of ammonia, spirits of nitre, stimulants and nutrients. He was in the habit of giving thirty-six ounces of whisky, and the same amount of beef tea in the twenty-four hours. In one or two cases out of the ninety-five treated he prescribed the *secale cornutum*.

In the *Medical Press and Circular* for June, 1867, we find the disease described as it occurred in Ireland; the treatment there generally adopted was brandy, beef tea, carbonate of ammonia, musk, ether, belladonna, turpentine, and in a few cases quinine and iron. From the reports of the cases this highly restorative treatment proved very unsuccessful. On the other hand, the French physicians bled their patients and had far greater success. Niemeyer, in his monograph on the disease, was also in favor of abstracting blood, and giving calomel freely: the latter, to say the least, is of doubtful efficacy except as a purgative.

In our city, in 1863 and '64, Dr. Ira Russell reported a number of cases in the *St. Louis Medical Journal* which occurred amongst the soldiers at Benton Barracks. About fifty cases had occurred at the time of his report, one-half of whom had died. The treatment was, during the period of collapse, alcoholic stimulants, capsicum and quinine, mustard emetics, and sinapisms to the surface. Opium seemed to exert a happy influence in controlling the delirium. After the reaction pillula hydrarg. and salines have been followed by good results, but as a general rule antiphlogistic treatment has been poorly borne. Cups to spine, iodide potassium and tonics, are the best remedies in the advanced periods of the disease. It is curious to observe how very seldom the *secale cornutum* or the iodide of potassium was prescribed in former times, although these seem now to be the favorite remedies with many practitioners. From the numerous reports of cases which I have examined I have been also surprised at the great differences of opinion with regard to the use of quinine and blood letting. Some believe that quinine is the main remedy, whilst others affirm that it never has a curative action, but often inflicts great injury, but I believe these discrepancies can all be reconciled when we reflect on the different types of the disease in different epidemics and in different localities. With regard to cupping, leeching and purgatives, the same contrariety of opinion prevails, and may be explained in the same manner, for antiphlogistics will be salutary or pernicious according to the form and type of the disease, and the constitutional stamina of the patients. As before intimated, there is a great uniformity in the different reports I have examined as to the signs, symptoms, course of the disease and its morbid anatomy. One fact stands out prominently in all the reports of the symptoms, and that is, the sudden apparent prostration of the patient; I say *apparent* prostration, for I contend that this symptom here is the result of the poisoned condition of the blood, its engorgement in the vessels of the cerebro-spinal system, and the resulting functional disorders of respiration, digestion, sanguification, etc. The great prostration so early existing in this disease is entirely different, especially as an indication of treatment, from the prostration resulting from loss of blood, from any wasting evacuation, or from prolonged bad health. In this disease the general debility is, in part, caused by a redundancy of certain matters in the blood, and by an engorgement or congestion of that diseased blood in the vessels of the cerebro-spinal system, whereby the nerves supplying the organs of respiration, circulation, digestion, etc., are seriously involved; whereas the debility existing from loss of blood, from profluvia, from long continued bad health, etc., results from want of nutritive elements and from a wasting of the tissues. Another fact which stands prominent in this disease and which is noted by all observers, is the expanded condition of the blood vessels of the cerebro-spinal system, and their engorgement with abnormally fluid, dark colored blood. Very often this is the only morbid condition found on post-mortem examination, although in most cases there is a superabundant exudation, sometimes fluid like pure serum, sometimes containing flocculi, and sometimes a semi-organized plastic material like butter, and again purulent lymph or pure pus. Now taking these prominent facts into consideration, the indications of treatment seem to be to endeavor to prevent that stasis or local engorgement if it has not already taken place, or to remove it if it has; to prevent exudation, or obviate and resist its evil results if it already exists; to restore the blood to its normal healthy condition; and to assist the natural vital processes so as to enable the system to bear up under the deteriorating influences of the malady. In those cases resembling pernicious intermittent or congestive fever, the algid cases of Dr. Condie, hot water baths containing salt and mustard, frictions with tincture of capsicum and camphor, and the internal use of sulpho-carbolate of sodium, sulphurous acid, sulphite of soda, permanganate and chlorate of potash, applying any of these which seems to agree best with the patient. These antiseptic remedies should correct the toxæmia existing, and with that view should be given freely and frequently, and in a chemically pure form. Small quantities should be prepared each day, so that they would be always fresh and unchanged. As soon as they can be administered with any prospect of happy action, iodide of potassium and the fluid extract of ergot should be given, the former to promote the absorption of the exudation, and the latter to constringe the expanded blood vessels. Quinine and a small quantity of opium will also do much good in these algid cases; the former will greatly assist the anti-toxæmic remedies, and the latter will prove beneficial in the first stage of the disease from its power in preventing exudation. Of course nutrients will be necessary, but they should be of a fluid form, such as beef essence, rich milk, cream, soft boiled eggs, etc., etc. The great difficulty in this form of the disease, is that the whole system is so completely overwhelmed by the toxic power of the malady, that no remedy has the time or the power to act, death occurring so suddenly, or the processes of organic life so completely in abeyance, that no effect is produced.

In the tetanic form of the disease, those cases which seem to be of a more inflammatory nature, besides the hot bath, in the first stage, cups or leeches to the nuchæ and down the spine, or behind the ears, will be of decided advantage. The form of purgative I have thought best, has been calomel and jalap, some prefer calomel and colocynth, or calomel and aloes, from the belief that the colocynth and aloes have the effect of acting on the lower intestines and as derivatives from the brain. As there is often great nausea, and consequently purgatives administered by the mouth may not be retained, in such conditions enemata of oil and turpentine, or decoction of aloes, may be given. As soon as the bowels are evacuated, the antiseptics should be freely given, and alternating with them the fluid extract of ergot, to resist the expansion and engorgement of the blood vessels. The tincture or fluid extract of the calabar bean has been recommended for the same purpose, but of that I have had no experience. I would rely on the cups and leeches, the purgatives, and the ergot of rye, to prevent the effusion of these tetanic or inflammatory cases, as I have witnessed severe coma to ensue after the use of opiates, but whether *propter hoc* or merely *post hoc*, may be a question to be decided in the future. Instead of the cups or leeches and purgatives in these cases, some recommend the veratrum viride, but from what I have experienced in my own cases, and from what I have read of the results of the treatment by others, I believe this powerful arterial sedative is contra-indicated in this disease. Gelseminum and digitalis have also been highly recommended by some, but I have had no experience with them in this affection. Quinine combined with opium in the very beginning of the disease, and without the opium in the commencement of convalescence, will be found a valuable remedy, but after the effusion has taken place, and as long as we have evidence of the existence of extensive exudation, this alkaloid may prove a dangerous medicine. In the very commencement of the attack opium may do good in preventing effusion, but in all other periods of the disease it is a perilous appliance. The great hyperæsthesia, cephalalgia and mental disturbance will be more safely remedied by venesection, leeches or cups, purgatives, free doses of the bromide of potassium, warm baths, and frictions with a liniment composed of chloroform and tinctures of opium, aconite root, capsicum and camphor. After exudation has taken place, the iodide of potassium will be found of essential service. I have witnessed numerous cases with slow intermittent pulse, deafness, slow breathing, irregular pupils, apathy and stupor, and in some partial paralysis, which happily recovered under the use of the iodide. Of course in these cases it would be well also to blister the nape of the neck, or over the mastoids.

In young children the bromide is often substituted for the iodide of potassium; in these young patients I am very decidedly opposed to opiates in any form of the disease, as coma will assuredly soon follow their administration. As before remarked, fluid nutrients should be frequently administered, and good ventilation and the best possible hygienic and sanitary measures should be sedulously carried out wherever this fearful scourge prevails.

I have cited the treatment adopted by different physicians in different epidemics, occurring in different countries, and from the comparative clinical statistics deducable from them, I am convinced that an opium treatment, a quinine treatment, or a stimulant and highly restorative treatment, are all incompatible with the true pathology of the disease. That an extreme antiphlogistic treatment would also be pernicious, I have not the least doubt; and I also feel convinced that veratrum viride, aconite, gelseminum and calabar bean, are hazardous remedies in this protean disease. If opium is to be used at all it should be in the very commencement of the attack; for the only reason that I can discover for its employment is its reputed powers of preventing serous exudation; and if it is given after the exudation has occurred, it will likely imperil the life of the patient, by encouraging the full development of the comatose condition. In a disease characterized as this is, by a congestion or engorgement of the blood vessels of the cerebro-spinal system, I cannot see the rationale of administering quinine, unless in cases complicated with malaria and intermittent in their character, or as a tonic in convalescence, when the congestion and exudation are both removed. If we carefully examine the results of a stimulating and restorative treatment, as detailed of the different epidemics in Massachusetts, in Pennsylvania, in Ohio, in Illinois, in Missouri, and in Ireland, we will be horrified at the great mortality. In these different places we can see the detailed reports of numerous cases of the tetanic form of the disease where the patient lived long enough to permit the action of medicine to take place, and yet the cases nearly all died under quinine, opium and stimulants. From sixty to seventy-five per cent. died in these epidemics, whereas the rational and scientific treatment now adopted—this treatment which is founded on a just recognition of the pathology and morbid anatomy of the disease—will save at least seventy per cent. of the cases.

To epitomize what we consider a rational and scientific treatment, we would say, that in the fulminant, siderant or blasting cases, and in those cases which run a rapid course, accompanied by purpuric eruptions, in other words, the variety which has been variously named cold plague, malum Egyptiacum, black tongue, etc., and the form denominated spotted fever or purpuric fever, putrid fever, etc., we would use hot salt and mustard baths, chlorate or permanganate of potash, sulphurous acid, sulph. carbolate of sodium, bisulphite of soda, etc., and afterwards quinine and phosphoric acid. Chemical physicians have quite recently suggested the cautious trial of the carbazotate of ammonia in these algid forms of the disease, giving one-fourth of a grain, in the form of a small pill, every four hours. In the tetanic form of the disease, we would rely on sinapisms, hot baths, leeches, cups, or venesections, ice to the head, frictions, free purgation, followed by opium, or opium and quinine in the commencement of the attack, the bromide and iodide of potassium, and the fluid extract of ergot; the bromide and ergot to be given before the exudation, and the iodide afterwárds. Quinine we would use, in all cases complicated with malaria, in the beginning of the disease, or as a tonic to assist in promoting a favorable convalescence after the exudation has been removed. To essay the mitigation of toxæmia, some of the antiseptic remedies alluded to may be advantageously administered during the early periods of the malady. Of course, the best possible sanitary and hygienic measures should be enforced, and the patient should be supported by fluid nutrients, and tonic medicines judiciously administered after the inflammatory symptoms have abated.—*St. Louis Medical and Surgical Journal.*

